# Aberrant calcium signaling and neuronal activity in the L271H *CACNA1D* (Cav1.3) iPSC model of neurodevelopmental disease

**DOI:** 10.1038/s41380-025-03429-8

**Published:** 2026-01-09

**Authors:** Marcel Tisch, Stefanie M. Geisler, Elisa Gabassi, Quirin Schlemmer, Miriam Lechner, Julia-Anna Ulz, Marta Suarez-Cubero, Laura De Gaetano, Angeliki Spathopoulou, Jörg Striessnig, Nadine J. Ortner, Katharina Günther, Petronel Tuluc, Frank Edenhofer

**Affiliations:** 1 Institute of Molecular Biology, Department of Genomics, Stem Cell Biology and Regenerative Medicine, Center for Molecular Biosciences, University of Innsbruck, Innsbruck, Austria; 2 Institute of Pharmacy, Department of Pharmacology and Toxicology, Center for Molecular Biosciences, University of Innsbruck, Innsbruck, Austria

**Keywords:** Neuroscience, Stem cells, Molecular biology

## Abstract

Voltage-gated calcium channels (VGCCs) are essential for neuronal excitability and synapse transmission as well as gene transcription regulation controlling cellular differentiation and survival. Recently, genetic variants in the *CACNA1D* gene, which encodes the α_1_-subunit of voltage-gated Ca_v_1.3 L-type Ca^2+^-channels, were linked to neurodevelopmental disorders, but their pathophysiological role on neuronal activity and development in a human background remains unknown. Here, we report the first functional characterization of a patient-derived iPSC-based disease model of the *CACNA1D* L271H variant. We observed that Ca_v_1.3 is the dominantly expressed L-type calcium channel isoform in neural progenitor cells (NPCs). NPCs expressing the L271H variant exhibit increased spontaneous calcium transients compared to the WT controls. Differentiated L271H-variant midbrain neurons show a more depolarized resting membrane potential and reduced excitability. Cortical organoids generated from the L271H-iPSCs contain fewer and smaller ventricular-like structures indicating impaired cellular organization. We identify spatial distortion of radial glial cell distribution and accelerated neuronal differentiation in patient-derived organoids as judged by premature intermediate progenitor cell and neuron emergence. Unbiased transcriptomic analysis revealed numerous dysregulated genes that according to gene ontology analysis were associated with “transcriptional regulation”, “CNS development”, and “neurogenesis” including *PTN, POU3F2, CNTN4* and *AUTS2*. These findings imply that disease-causing Ca_v_1.3 variants alter ion homeostasis, result in aberrant neuronal function and distort human neurodevelopment, contributing to the complex disease phenotype observed in patients with high-risk *CACNA1D* variants.

## Introduction

Voltage-gated calcium channels (VGCCs) represent a class of ion channels found in the plasma membrane of excitable cells that tightly control the calcium influx crucial for proper development and function. Consequently, pathologic alterations in VGCC activity cause human disease [1]. The L-type VGCC Ca_v_1.3 is widely expressed throughout the human body including the brain, sinoatrial node, cochlea, and endocrine tissues such as the adrenal gland or the pancreas [2, 3]. In the mammalian brain, Ca_v_1.3 channels play pivotal roles in the modulation of action potentials (APs) and cellular excitability, stabilization of pacemaker activity in midbrain dopaminergic neurons, and the regulation of synaptic plasticity and dendritic spine morphology [3–8]. Moreover, Ca_v_1.3-mediated intracellular calcium signals contribute to excitation-transcription coupling, shaping the transcriptome of excitable cells [9, 10]. Thereby, they control adult neurogenesis, impacting proliferation, migration, differentiation of neural progenitor cells (NPCs) and synaptogenesis of newly formed neurons influencing critical aspects of neuronal subtype specification, network assembly and brain development [11–15].

Previous studies have linked de novo missense variants in *CACNA1D*, the gene encoding the central pore-forming α_1_ subunit of Ca_v_1.3, to a rare neurological and endocrine disorder [8, 16–20]. To date, a total of at least 17 pathogenic germline *CACNA1D* variants have been reported which recurrently present similar clinical manifestations, including severe primary aldosteronism and hyperinsulinemic hypoglycemia, facial dysmorphisms, seizures, auto-aggressiveness, developmental delay and autism spectrum disorder (ASD), indicating a multisystem role for this gene [21, 22]. The changes in Ca_v_1.3 channel biophysical properties induced by the missense variants have been characterized using heterologous overexpression in tsA201 cells and revealed typical and variant-specific gain-of-function phenotypes [21]. Two recently published mouse models involving activity-enhancing *CACNA1D* variants, i.e. A749G or I750M, confirmed the pathogenicity in vivo, as these mice recapitulated clinical phenotypes observed in humans [8, 23]. Mice carrying the A749G *CACNA1D* variant display increased neuronal excitability in selective striatal dopaminergic neuron populations, highlighting their crucial role for neuronal function [8]. A young female patient with the de novo L271H *CACNA1D* variant has been reported with hyperinsulinemic hypoglycemia, primary hyperalderosteronism, and muscular hypotonia [24]. Further manifestations included delayed speech development and behavioral symptoms, suggesting a severe neurological phenotype. Exome sequencing pinpointed the heterozygous c.812 T > A (GRCh38) variant in exon 6 of the *CACNA1D* gene as the underlying pathogenic variant, absent in the patient’s parents [24]. Like other pathogenic *CACNA1D* variants, L271H Ca_v_1.3 channels showed altered channel gating, including a typical shift of the voltage-dependence of gating to more hyperpolarized potentials during overexpression in non-neural tsA201 cells [25]. However, the cellular and molecular consequences of *CACNA1D* (Ca_v_1.3) variants in more relevant human neural models remain to be investigated.

Here we describe the first patient-derived model of a *CACNA1D* (Ca_v_1.3) variant, employing iPSC-derived neural progenitor cells (NPCs), dopaminergic neurons and cortical organoids from the patient carrying the germline L271H substitution. We observed that Ca_v_1.3 is the dominantly expressed L-type VGCC isoform in NPCs on a transcriptomic level, preceding the expression of the other brain L-type isoform Ca_v_1.2 during neuronal maturation. While patient-derived NPCs exhibit increased spontaneous calcium transients, differentiated dopaminergic neurons show reduced spontaneous firing and hypo-excitability, linked to a depolarized resting membrane potential. Unbiased transcriptomic analysis revealed numerous dysregulated genes that according to gene ontology analysis are associated with processes related to “transcriptional regulation”, “CNS development”, and “neurogenesis”. These findings suggest that Ca_v_1.3 dysregulation plays a critical role in early neurodevelopment by disruption of ion homeostasis, highlighting its impact on early brain development and cellular organization.

## Results

### The Ca_v_1.3 L271H variant enhances spontaneous calcium transients in NPCs

**Fig. 1:**
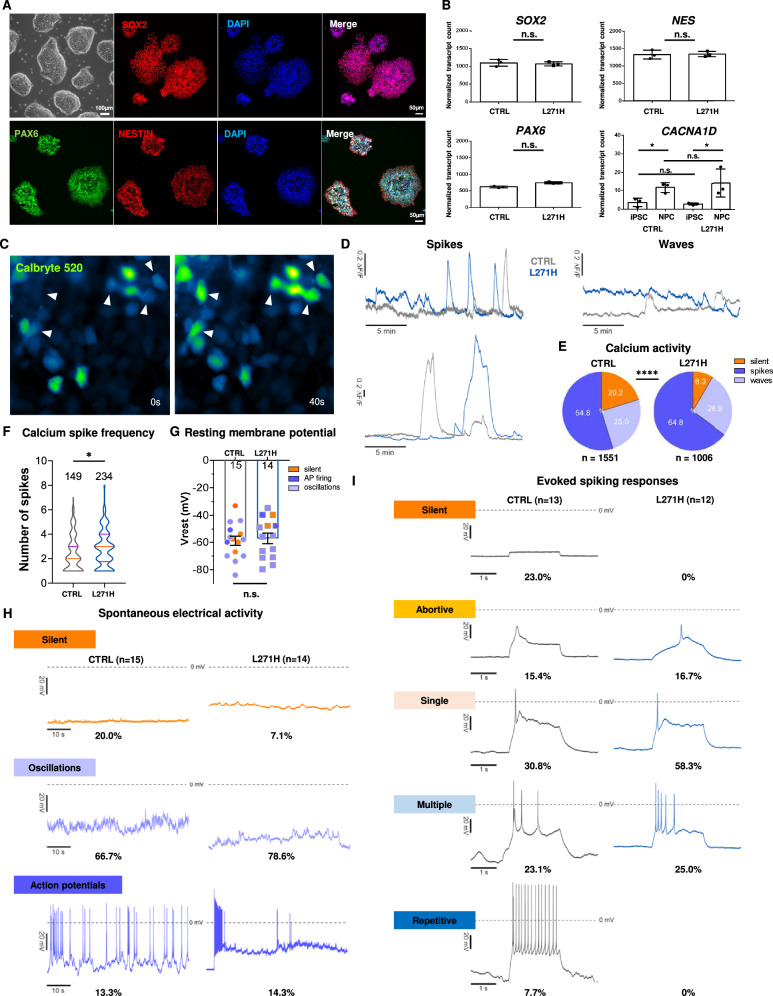
Validation and functional analysis of patient-derived NPCs. **A** iPSC-derived NPCs express established NPC markers SOX2, NESTIN and PAX6. **B** Expression levels of control and L271H NPC in bulk RNAseq data. N = three biological replicates. * = P < 0.05 **C** To measure Ca^2+^ transients in control and patient-derived NPCs expressing the L271H variant, cultures were loaded with Calbryte 520 AM Ca^2+^ indicator. Fluorescent signals of single cells were acquired during a 20 min recording period (Supplementary video 1). **D** Representative Ca^2+^ transients of control and L271H NPCs exhibiting calcium waves (slow-rising events, ∆F/F < 0.2) or spikes (fast rising events, ∆F/F > 0.2). **E** Quantification of intracellular Ca^2+^ activity patterns, showing an increase in the population of cells displaying Ca^2+^ waves or spikes in L271H cultures. Chi-squared test with **** = P < 0.0001. **F** Quantification of Ca^2+^ spike numbers during 20 min recordings. * = P < 0.05. **G** Estimated resting membrane potential of silent cells (orange), cells with subthreshold membrane oscillations (light purple) and cells displaying action potential firing (dark purple). **H** Representative traces of spontaneous electrical activity patterns and quantification of firing behaviors. Color code as in G. **I** Starting from a holding membrane potential of −70 mV, action potential firing was elicited by 2 s depolarizing current injections in 1 pA increments. Representative traces depict induced electrical activity and quantification of firing behaviors (categories: silent = no AP, abortive = small amplitude & slow upstroke, single = single AP with overshoot, slope, multiple = < 10 APs, repetitive = > 10 APs). N = Number of cells.

Healthy control or patient iPSCs carrying a heterozygous c.812 T > A (GRCh38) variation in exon 6 of the *CACNA1D* gene were used for the differentiation into NPCs, which mimics the onset of neurulation and the establishment of neuroepithelial progenitors [26, 27]. To control for genetic background and clonal variability, we used five subclones derived from three independent healthy control iPSC lines and three subclones of the L271H patient line. The obtained NPCs express established NPC markers, including SOX2, PAX6 and NESTIN (*NES*) on both the protein and mRNA level (Fig. 1A, B). Comparable levels of these markers were detected in both the control and patient-derived cells, suggesting successful neural induction (Fig. 1B). Upon NPC differentiation, an increase in *CACNA1D* expression was observed in both genotypes (CTRL: 3.18-fold mean increase, L271H: 4.97-fold mean increase, adj. P < 0.05), as compared to the parental iPSCs (Fig. 1B). Notably, there was no change in the expression of the *CACNA1C* gene, which encodes the Ca_v_1.2 VGCC α_1_-subunit (Suppl. Figure 1A, B), the dominant L-type VGCC isoform in the adult mammalian brain [28]. This finding indicates an important role of Ca_v_1.3 at this early stage of human neurodevelopment.

To probe for potential alterations of cellular function caused by Ca_v_1.3-L271H channels, spontaneous calcium transients were recorded in live NPCs from 3 biological replicates by measuring the calcium-dependent change in fluorescence intensity of the Calbryte 520AM calcium indicator. Imaging was performed in a blinded manner on all five independent subclones of three iPSC lines from healthy controls and from three independent clones derived from the patient’s peripheral blood mononuclear cells (PBMCs) (CTRL: n = 1551 cells, L271H: n = 1006 cells). The recorded traces were used to group the activity of individual cells according to previously published criteria [29] by categorizing traces showing no discernible calcium activity as silent, transient calcium oscillations (slow rising phases, ∆F/F < 0.2) as waves and calcium peaks (fast-rising phase events, ∆F/F > 0.2) as spikes (Fig. 1C, D, Suppl. Video 1). Cultures derived from the patient’s iPSCs exhibited significantly less silent cells (8.3%) as compared to the controls (20.2%). Additionally, we observed an increase in the population of cells displaying spontaneous calcium transients in the form of waves (CTRL: 25.0% vs. L271H: 26.9%) and spikes, respectively (CTRL: 54.8% vs. L271H: 64.8%) (Fig. 1E, Chi-squared test, P  < 0.0001). Additionally, patient-derived NPCs showed significantly more calcium spikes over a 20 min recording period compared to controls (Median number of spikes = CTRL: 2, 95% Cl = 2.2, 2.7; n = 149 cells vs. L271H: 3, 95% Cl = 2.6, 3.0; n = 234 cells; P = 0.02) (Fig. 1F). To explore to which extent these significant differences in the activity of calcium transients are reflected by altered excitability, we measured the electrical activity of NPCs across four biological replicates using whole-cell current-clamp recordings, as previously described [8, 30, 31]. The resting membrane potential (RMP) was comparable between the control and patient-derived cells (CTRL: V_rest_ = −58.97 ± 3.3 mV, n = 15 cells vs. L271H: V_rest_ = −57.2 ± 3.9 mV, n = 15 cells; P = 0.7) (Fig. 1G). Similarly, the proportion of cells displaying spontaneous electrical activity either in form of subthreshold membrane oscillations (approx. 5 to 10 mV) or AP firing did not significantly differ between genotypes (CTRL: 80% vs. L271H: 93%; P = 0.48) (Fig. 1H). To assess firing capacity we measured to which extent APs could be similarly evoked in control and patient-derived NPCs by applying stepwise depolarizing current injections while clamping the membrane potential at −70 mV. APs could readily be evoked in a large fraction of control and patient-derived NPCs, with no significant difference in the proportion of cells displaying mature APs (CTRL: 8 of 13 vs. L271H: 10 of 12 cells with mature APs displaying an overshoot above 0 mV; P = 0.48) (Fig. 1I).

Taken together these data suggest that although Ca_v_1.2 is the predominant L-type VGCC isoform in the adult brain (~90%) [28], at the NPC stage, Ca_v_1.3 is the dominantly expressed L-type calcium channel. Moreover, the Ca_v_1.3 L271H variant causes a significantly increased spontaneous calcium transient activity in NPCs, consistent with a role in intracellular calcium signaling. This phenotype is not accompanied by changes in intrinsic excitability, however, suggesting an early, non-excitability driven role of Ca_v_1.3 L-type channels in human neurodevelopment.

### Patient-derived midbrain neurons display reduced spontaneous AP firing and hypo-excitability

**Fig. 2:**
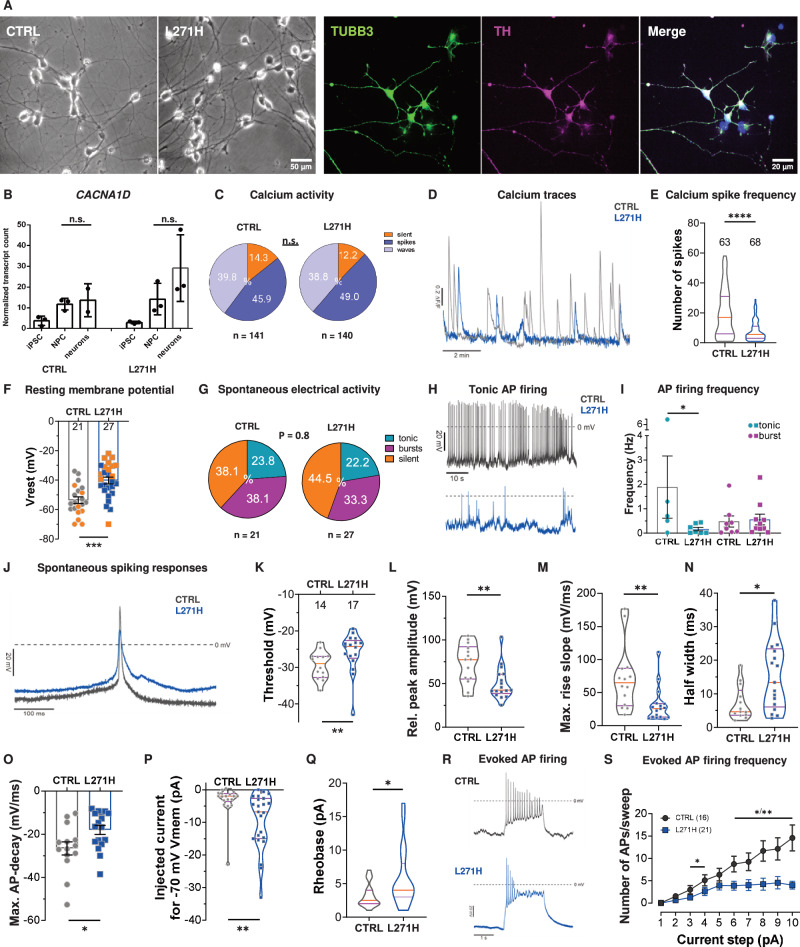
Validation and functional analysis of patient-derived midbrain neurons. **A** Representative images of the obtained neuronal cultures and immunostaining for TUBB3 and TH after 30 days of differentiation. Nuclei counterstained with DAPI. **B** Expression levels of *CACNA1D* in the control and patient-derived neurons throughout in vitro differentiation. N = three biological replicates derived from bulk RNAseq data. **C** To measure Ca^2+^ transients in control and patient-derived neurons expressing the L271H variant, cultures were loaded with Calbryte 520 AM Ca^2+^ indicator. Fluorescent signals of single cells were acquired during 10 min recordings (Supplementary video 2). Quantification of intracellular Ca^2+^ activity in control and L271H neurons. Cells were categorized as silent (orange, baseline fluorescence), displaying Ca^2+^ events in form of waves (light purple, slow-rising events) or spikes (dark purple, fast-rising events). **D, E** Representative traces of Ca^2+^ spikes and quantification of spike numbers per 10 min recording. **F** Estimated resting membrane potential of control and L271H neurons. ** = P < 0.01. Orange = silent cells. Grey/blue = active cells. **G** Quantification of spontaneous electrical activity after categorizing action potential-firing behavior as silent, tonic or burst firing. **H** Representative 1 min recordings of control and patient-derived cells showing tonic action potential firing. **I** Quantification of firing frequency of cells exhibiting tonic or burst firing during spontaneous electrical activity. **J** Representative traces of spontaneous action potentials, displaying different action potential shapes in L271H neurons. **K**–**O** Quantification of spontaneous action potential parameters, including the threshold, relative peak amplitude, maximal rise slope, half width and maximal decay slope. * = P < 0.05, ** = P < 0.01 **P** Quantification of injected current required to clamp the cells at a membrane potential of −70 mV. ** = P < 0.01 **Q** Rheobase needed to evoke action potentials in control and L271H neurons. * = P < 0.05 **R** Representative traces of action potentials evoked upon current injection (2 s, 6 pA injection). **S** Quantifications of action potential numbers per sweep in relationship to the injected current * = P < 0.05, ** = P < 0.01, *** = P < 0.001.

Ca_v_1.3 channels play an essential role in midbrain dopaminergic neuron function [8, 32–34] making them an ideal model system to investigate the role of mutant Ca_V_1.3 channels in mature neurons. First, we subjected CTRL and L271H NPCs to a targeted midbrain neuronal differentiation paradigm [35], resulting in up to 60% TH-positive neurons. To validate their midbrain dopaminergic character we additionally confirmed expression of EN1 and DDC at protein and *FOXA2, EN1* and *LMX1B* at RNA level (Fig. 2A, Suppl. Figure 1C–E). While the transcriptomic data demonstrate a small increase in *CACNA1D* expression levels compared to the NPC state in both CTRL and L271H lines (CTRL: 1.2-fold mean increase, L271H: 2.1-fold mean increase, not significant, adj. P > 0.05) (Fig. 2B), *CACNA1C* (Ca_v_1.2) became the dominant L-type VGCC isoform on the mRNA level (CTRL: 20.3-fold, L271H: 57.2-fold mean increase from NPCs to neurons, adj. P < 0.001) (Suppl. Figure 1A, B). Unlike in the NPC state, the number of cells showing the different intracellular calcium activity patterns (silent, spikes, waves) was not significantly different between control and patient-derived midbrain neurons over a 10 min recording period (CTRL: n = 141 cells, L271H: n = 140 cells) (Fig. 2C, Suppl. Video 2). In both genotypes a similar percentage of cells exhibited calcium spikes (CTRL: 45.9% vs. L271H: 49.0%,) with overall increased frequency compared to the NPCs (Fig. 1F & Fig. 2E). However, the number of calcium spikes per 10 min recording within individual neurons was significantly decreased in the patient-derived cells (Fig. 2D, E) (Median number of spikes = CTRL: 1.7 spikes/min; n = 63 cells vs. L271H: 0.55 spikes/min, n = 68 cells; P < 0.0001).

To assess whether the L271H substitution alters the electrical activity of the differentiated neurons, we recorded the spontaneous AP firing of individual cells in current-clamp mode across four biological replicates in a blinded manner. The electrical activity of dopaminergic midbrain neurons is characterized either by tonic single spike firing or AP-bursts showing a strong spike-frequency adaptation separated by electrically silent periods [36, 37]. The proportion of cells exhibiting spontaneous electrical activity was comparable between genotypes, with similar distributions of silent, tonically firing, and burst-firing cells (silent: CTRL: 38.1% vs. LH: 44.5%; tonic: CTRL:23.8% vs. LH: 22.2%; burst: CTRL: 38.1% vs LH: 33.3%; P = 0.8) (Fig. 2F, G). However, tonically firing L271H neurons displayed a markedly reduced firing frequency compared to controls (CTRL: 1.89 ± 1.3 Hz, n = 5; L271H: 0.17 ± 0.07 Hz, n = 7; P < 0.05) (Fig. 2H, I), which was linked to a significantly more depolarized resting membrane potential (RMP) in patient-derived cells (CTRL: −53.7 ± 2.2 mV, n = 21 cells; L271H: −40.3 ± 2.2 mV, n = 27 cells; P < 0.001) (Fig. 2F). Consistent with a more depolarized RMP and reduced electrical activity, the APs of the patient-derived cells exhibited a significantly more depolarized threshold of AP firing, a reduced relative peak amplitude, a decreased maximal rise slope and a decreased maximal decay slope compared to the respective control cells (Fig. 2J–O).

To test whether patient-derived neurons display altered excitability independent of the differences in RMP, we clamped the RMP at −70 mV and applied stepwise current injections of increasing amplitude. APs could be evoked in all cells upon current injections, including the cells that did not show spontaneous activity. However, to hyperpolarize the cells at −70 mV, significantly more negative current had to be injected into the patient-derived neurons compared to the control (CTRL: I_hyp_ = −3.4 ± 1.2 pA, n = 18 cells vs. L271H: I_hyp_ = −9.5 ± 1.8 pA, n = 23 cells; P < 0.01) (Fig. 2P). The stepwise increased current injections induced an electrical activity in cells of both genotypes, but the patient-derived cells required more current to trigger AP firing (CTRL: I_reo_ = 3.2 pA, 95% Cl = 2.2, 4.1; n = 16 cells vs. L271H: I_reo_ = 5.7 pA, 95% Cl = 3.7, 7.7; n = 21 cells; P < 0.05) (Fig. 2Q). Although multiple action potentials were observed in both conditions at each current step injection, the patient-derived neurons typically went into depolarization block earlier (Fig. 2R) and eventually displayed an overall reduced firing frequency at higher current injections (at 6 pA injections: CTRL: N = 8.8 ± 1.8 APs/sweep, n = 16 cells vs. L271H: N = 4.0 ± 1.0 APs/sweep, n = 21 cells; P < 0.05) (Fig. 2S). Similar to the changes in AP shape observed during spontaneous activity, evoked APs fired by the patient-derived cells also exhibited an increased threshold, a reduced relative peak amplitude and a decreased maximal rise slope as compared to the respective control (Suppl. table 1). Taken together, our data demonstrate that the *CACNA1D* (Ca_v_1.3) L271H variant causes reduced spontaneous pacemaker firing and hypo-excitability in midbrain neurons, which may contribute to the neurological phenotype observed in the patient.

### The L271H variant disrupts NPC self-organization during neuronal differentiation

**Fig. 3:**
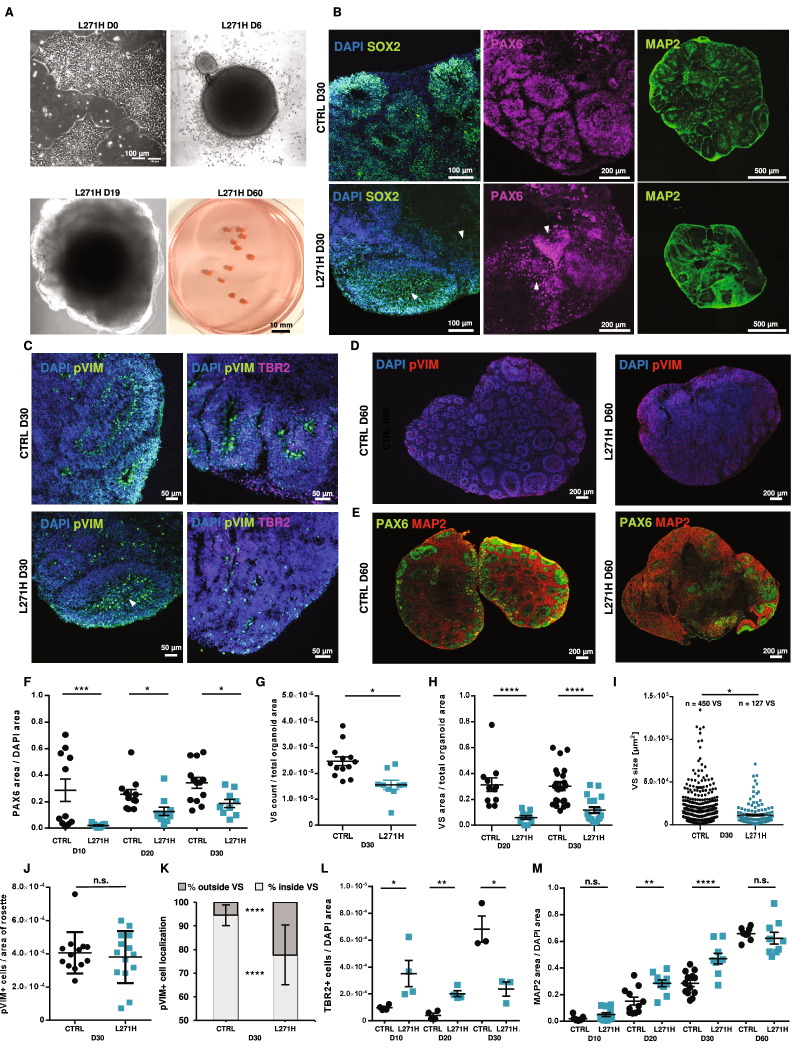
Generation of cortical organoids and image-based quantification. **A** Representative images of the L271H cortical organoids throughout different stages of generation displaying the initial culture of iPSCs, the aggregated EBs and the growing organoids. **B**, **C** Immunostainings of SOX2, PAX6, MAP2, pVIM and TBR2 in the 30-day old organoids highlighting neural progenitors, radial glial cells, intermediate progenitor cells (IPC). Arrow heads indicate disorganized ventricular-like structures (VS). **D**, **E** Immunostainings of pVIM and MAP2 in the day 60 organoids staining VS and neurons. **F** Image-based quantification of PAX6 area per total DAPI area of the organoid at day 10, 20 and 30 of differentiation. N = 9–12 organoids from 3 batches. **G**–**I** Quantification of VS area, size and count. N = 9–12 organoids from 3 batches. **J** Quantification of pVIM+ radial glial (RG) cells per VS area. N = 18 (CTRL) vs. 20 (L271H) VS from 3 organoids. **K** Image-based quantification of RG localization inside or outside of VS. N = 18 (CTRL) vs. 20 (L271H) VS from 3 organoids **L** Image-based quantification of TBR2+ intermediate progenitor cells (IPC) per total DAPI area at day 10, 20 and 30 of organoid generation. **M** Quantification of MAP2 area over total DAPI area in 10-, 20-, 30- and 60-day old organoids. Unpaired t-test, * = P < 0.05, ** = P < 0.01, *** = P < 0.001, **** = P < 0.001.

Next, we studied the impact of the Ca_v_1.3 L271H variant on early processes of cellular rearrangement and neuronal differentiation. To this end, CTRL and patient-derived iPSCs were subjected to 3D cortical organoid generation [38–40]. Aggregation of plated cells resulted in the formation of cell spheres, in which neural induction was achieved, for both control and patient-derived lines (Fig. 3A). After 10, 20, 30 and 60 days of in vitro differentiation, the developing organoids were harvested and analyzed (three organoids per condition per timepoint from three independent batches). While cell aggregate size did not differ significantly in the early stages (D3-D10) of organoid generation, by day 20 the L271H organoids appeared smaller in overall size (Suppl. Figure 2A). Successful neural induction was observed in both conditions at day 30, as indicated by the presence of both SOX2- and PAX6-positive NPCs as well as MAP2-positive neurons (Fig. 3B). Moreover, we observed pVIM-positive radial glial (RG) cells and EOMES/TBR2-positive intermediate cells (IPCs) cells (Fig. 3C, D, Suppl. Figure 3H), While canonical NPC and neuronal markers were generally present in organoids from both genotypes, a striking difference was observed in the formation of ventricle-like structures (VS) and the organization of adjacent neural progenitor cell domains (arrowheads in Fig. 3B, C). To assess this phenotype in more detail we performed quantifications on 9–12 organoids from three independent batches per condition, revealing significantly less PAX6-positive NPCs in the L271H organoids at all time points up to day 30 (Fig. 3F). Moreover, while RG cells of the control samples were localized into distinct highly polarized VS surrounding an apical lumen, patient-derived cells largely failed to self-organize into those characteristic structures, as indicated by an overall 1.6-fold lower VS count (Fig. 3G). Additionally, we quantified a reduced VS area and a smaller VS size (CTRL mean: 1.62 ×104 μm^2^; L271H mean: 1.15 ×104 μm^2^) (Fig. 3H, I, Suppl. Figure 2B–D). While the ratio of pVIM-positive RG per VS area remained unchanged between control and L271H organoids (Fig. 3J, Suppl. Figure 2E), a significant shift in RG distribution throughout the whole organoid was observed (Fig. 3K, Suppl. Figure 2F, G). Instead of being restricted to the inner lining of the VS, as seen in the control organoids, a large fraction of RG (mean: 22.3% of pVIM+ cells) were found outside of the VS and sparsely spread all throughout the L271H organoids as compared to the control (mean: 5.5%) (Fig. 3K, Suppl. Figure 2F, G). Additionally, significant differences in the development of TBR2-positive IPCs were observed. While day 10 and day 20 L271H organoids showed a significantly higher proportion of IPCs, their number was significantly reduced by 65.4% at day 30 as compared to the IPC levels detected in the control organoids (Fig. 3L, Suppl. Figure 2H). Notably, the abundance of MAP2-positive neurons was also affected in the L271H condition (Fig. 3B, E, M). Quantification of MAP2 revealed a significantly increased MAP2 area on day 20 and 30 (Fig. 3M). Interestingly, by day 60 of organoid generation no difference in MAP2+ neuron abundance was detected (Fig. 3M). In conclusion, these results suggest an impairment of cellular organization in the L271H organoids, required for the formation of VS, rather than discrepancies in the overall differentiation potential of the patient-derived cells. Particularly, changes in PAX6-positive NPC population size, altered timing of IPC appearance (i.e. earlier appearance and earlier depletion), but maintained neurogenesis, suggest an accelerated neuronal differentiation in the L271H samples.

### The Ca_v_1.3 L271H variant causes upregulation of neurodevelopmental genes

**Fig. 4:**
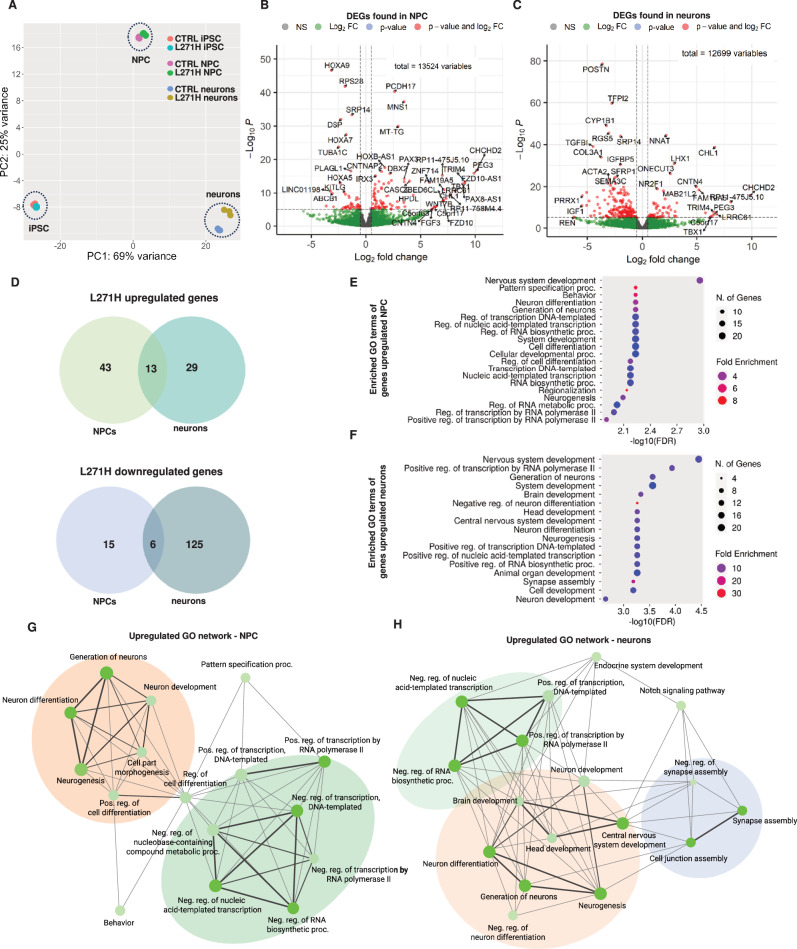
Transcriptomic analysis of patient-derived NPCs and neurons. **A** PCA plot of control and patient-derived iPSCs, NPCs and midbrain neurons indicating clear separation of the samples over the course of in vitro differentiation. Principal component (PC) 1 explains 69% of the total variance, whereas PC2 explains 25% of the total variance. N = Three biological replicates each. **B**, **C** Volcano plot of differentially expressed genes among control and L271H NPCs or neurons. Cutoffs: log2FC > |0.5| and adj. p-values < 1*10^−5^ **D** Venn diagram of overlapping up- or downregulated genes found in the patient-derived NPCs and neurons. **E**, **F** GO term analysis of upregulated genes in patient-derived NPCs or neurons. FDR = false-discovery rate **G**, **H** Networks of GO terms associated with upregulated genes found in the L271H NPCs and neurons. Colors highlight groups of similar GO-terms.

Since L-type VGCCs and electrical activity can regulate excitation-transcription coupling, we assessed consequences of the Ca_v_1.3 L271H variant at transcriptome-wide level in an unbiased manner. To this aim, we performed bulk RNA sequencing on the patient-derived iPSCs, NPCs and midbrain neuronal cultures (three independent biological replicates per condition) to investigate potential transcriptomic changes caused by the *CACNA1D* (Ca_v_1.3) L271H variant. Principal component analysis (PCA) revealed a clear separation of the different cell types along the PC1 axis, which explains 69% of the variance (Fig. 4A). Interestingly, control and patient-derived samples clustered in proximity at the iPSC stage but exhibited increasing separation in the NPC and neuronal samples. Together, these findings indicate that transcriptomic changes between the conditions become more dominant as neural differentiation progresses (Fig. 4A). This might be the consequence of increasing *CACNA1D* expression upon neural induction, as compared to low expression levels in the iPSCs (Fig. 1B, Fig. 2B). To study the underlying transcriptomic alterations in more detail, differential gene expression analysis was conducted. This led to the identification of 56 significantly upregulated and 21 downregulated genes between patient-derived and control NPCs (Fig. 4B, Suppl. Table 2, log_2_FC > |0.5|, adj. P < 1 × 10^−4^). Similarly, a total of 41 genes were found upregulated in the patient-derived midbrain neurons, whereas 131 genes were significantly downregulated when compared to the control midbrain neurons (Fig. 4C, Suppl. Table 2, log_2_FC > |0.5|, adj. P < 1 × 10^−5^). Comparative analysis of the differentially expressed genes (DEGs) indicated that, although there is a small overlap among the upregulated (13 genes) or downregulated (6 genes) genes found in both the NPCs and midbrain neurons, the majority of DEGs are unique to the respective cell types (Fig. 4D). However, when performing gene ontology (GO) analysis on the DEGs for both cell types, many of the same biological processes were affected by the upregulated genes observed in the Ca_v_1.3 L271H variant whereas the GO terms for the downregulated DEGs were less conclusive (Fig. 4E–H, Suppl. Figure 2). In both conditions this included upregulated genes associated with “regulation of transcription”, especially “involving RNA polymerase II” suggesting an impact on excitation-transcription coupling, including *AUTS2*, *MEIS2* and *POU3F2*. Additionally, processes related to “neurogenesis”, “neuron development” and “neuron differentiation” were significantly enriched among the upregulated DEGs. In the NPCs this included genes such as *PTN* and *AUTS2*, which have previously been described as neurodevelopmental regulators and risk genes for neurodevelopmental disorders such as ASD [41–46], while in the neurons other developmental regulators such as *MEIS2* and *POU3F2* (BRN2) underlie the respective GO terms. Notably, GO terms associated with synapse assembly were only enriched in the neuronal samples, suggesting that the Ca_v_1.3 variant may in fact cause transcriptomic changes influencing essential pathways of neurogenesis through the upregulation of synapse formation regulators, such as *ROBO2, SLIT1* or *DNER*. In order to assess whether altered Ca_v_1.3 activity results in dysregulation of other ion channels, we investigated relevant voltage-gated sodium, potassium and calcium channel subunits (Suppl. Table 3). In fact, no significant transcriptomic differences in any of those channel-encoding genes were detected except for *CACNA2D1*. This gene encodes the auxiliary α_2_δ_1_ subunit of VGCCs, one of the four α_2_δ isoforms regulating pre- and postsynaptic functions [47], and was significantly upregulated in the patient-derived midbrain neuronal cultures. Taken together, the transcriptomic changes observed in the patient-derived samples indicate alterations in the expression patterns of both RNA polymerase II-dependent transcription and neurodevelopmental programs. These alterations could explain the differences seen in the 3D model of early neurodevelopment, described above. Intriguingly, several of the dysregulated genes have previously been associated with ASD and therefore might be potential underlying drivers of the clinical manifestations seen in the patient.

## Discussion

Increasing evidence suggests that *CACNA1D* (Ca_v_1.3) variants cause developmental disorders with broad neurological and endocrine symptoms in a constantly rising number of affected patients [2, 21, 22]. Although characteristic pathogenic gating changes have been shown in mice and non-neural human models, a detailed understanding of how this translates into altered cellular signaling affecting human neurodevelopment and brain function is unknown [2, 21]. Stem cell-based disease models derived from patients carrying disease variants of interest can help shed light on these fundamental questions. For instance, iPSC-based disease models for *CACNA1C* (Ca_v_1.2) variants underlying the pathology of Timothy syndrome were instrumental for a deeper understanding of the functional role of Ca_v_1.2 in neurodevelopment and disease [48–51]. Particularly, iPSC-derived neural models containing the G406R substitution within the L-type VGCC Ca_v_1.2 α_1_-subunit isoform have been reported to replicate cardiac arrhythmias and neurodevelopmental abnormalities [49, 50, 52, 53]. However, no comparable model has been reported for *CACNA1D* (Ca_v_1.3) variants thus far.

Here, we have chosen the *CACNA1D* (Ca_v_1.3) L271H variant to generate iPSC-derived human neural models based on its association with a complex pathology and its putative molecular mode of neuronal dysfunction. The L271H substitution is located in the S4-S5 linker, a channel part responsible for the coupling of the voltage-sensor to pore-opening. Based on analyses in tsA201 cells, the L271H substitution strongly affects voltage-dependent channel gating enabling increased calcium channel function at subthreshold potentials similar to most other known pathogenic *CACNA1D* variants [21, 22, 25]. While tsA201 cells provide a convenient system for heterologous overexpression of ion channel variants and are useful for basic characterization of channel properties, they have significant limitations in modeling the complexity of neuronal electrophysiology. Their non-neuronal origin, lack of synaptic components, and inability to mimic neuronal excitability and plasticity make them less ideal for studies focused on neurodevelopmental disorders. To overcome these limitations, our study employs iPSC-derived neuroepithelial progenitors, differentiated midbrain neurons, and cortical organoids, allowing us to explore the stage-specific impact of the L271H variant on calcium signaling, membrane excitability, and neural differentiation. We demonstrate that approximately 80% of the control NPCs exhibit detectable spontaneous intracellular calcium transients, either in the form of calcium oscillations or spikes. We conclude, that control of intracellular calcium signaling is the dominant role of the Ca_v_1.3 channel during this developmental period. In fact, our transcriptomic and qPCR results show that Ca_V_1.3 is the dominantly expressed L-type VGCC isoform at the NPC mRNA level, preceding the expression of Ca_V_1.2 channels, which comprise the major isoform in differentiated neurons and adult brain [28].

Additionally, we demonstrate that L271H-variant NPCs exhibit significantly increased spontaneous calcium activity, without corresponding changes in excitability, suggesting altered calcium homeostasis. As calcium imaging reflects a combination of subthreshold depolarizations, spontaneous network-driven activity, calcium channel function, and intracellular buffering dynamics, our findings indicate that Ca_V_1.3 dysfunction at the NPC stage primarily disrupts calcium signaling rather than electrical firing properties. This phenotype may reflect an early, non-excitability-driven role of Ca_V_1.3 L-type calcium channels in neurodevelopment, such as regulating gene expression, proliferation, and cell fate- possibly contributing to the observed morphological and transcriptomic defects seen in the differentiated 2D cultures and cortical organoids. In contrast to NPCs, differentiated midbrain neurons carrying the Ca_V_1.3 L271H variant exhibit reduced calcium transients and impaired excitability, including a significantly depolarized RMP and altered action potential properties. These findings suggest that Ca_V_1.3 channels gain a more central role in regulating cellular excitability as neurons mature. Notably, neurons carrying the L271H variant require more current injection to maintain their membrane potential at −70 mV, suggesting a persistent depolarizing current. We did not detect significant differences in voltage-gated sodium channel expression on the mRNA level between the control or patient-derived neurons, which could account for this effect. However, given the known gating changes of the L271H variant in heterologous cells, specifically, the negative shift in the voltage-dependence of activation, we hypothesize that increased window current activity at subthreshold voltages [25] contributes to the observed membrane depolarization. Consequently, the continuous depolarization of the membrane potential may result in voltage-dependent inactivation of other ion channels important for proper neuronal excitability and AP generation in dopaminergic midbrain neurons, such as voltage-gated sodium channels [54]. This would also explain the increased action potential threshold, decreased maximal rise slope and overall reduced firing frequency exhibited by the patient-derived midbrain neurons. Future studies should therefore investigate the cellular mechanisms by which Ca_V_1.3 L271H variants affect midbrain neuron excitability, for instance by measuring differences in ionic conductance contributing to DA neuronal excitability (e.g. Ca^2+^, K^+^ [55], Na^+^ [56]).

Interestingly, in mice carrying the A749G *CACNA1D* variant, dopaminergic midbrain neurons in the medial substantia nigra displayed enhanced pacemaker activity, confirming an impact of aberrant Ca_v_1.3 function on their firing behavior [8]. The opposite effect on excitability, compared to the iPSC-derived midbrain neurons studied here, might be due to the distinct gating profiles of the two *CACNA1D* variants, the different identity or maturation state of the neurons, or may reflect potential interspecies differences. In the human brain these functional alterations could potentially cause discrepancies in the firing behavior or propagation of electrical signals among neurons and might thereby negatively contribute to the neurological phenotype the patient has been diagnosed with. Our investigation to which extent the *CACNA1D* L271H variant results in dysregulation of other ion channels that could potentially explain the functional electrophysiological differences revealed no significant changes except upregulation of *CACNA2D1*. This gene encodes the α2δ-1 auxiliary subunit of VGCCs and its dysregulation has also been associated with ASD [47, 57]. Our observation of increased α2δ-1 expression suggests compensatory modulation of channel gating and downstream signaling, which needs to be investigated in future studies.

In addition to the functional alterations observed in the patient-derived NPCs and midbrain neurons, the *CACNA1D* L271H variant also had neurodevelopmental consequences in vitro. Our results demonstrate a reduction in organoid size in the L271H samples, concomitant with a significantly reduced PAX6-positive NPC progenitor pool size, as well as a disorganization of RG and ventricular-like structures. Finally, we detected a premature appearance of TBR2-positive IPCs followed by a significant depletion of these cells at later stages. Interestingly, the dynamics of MAP2+ neuron generation mirror this development, as a significantly higher abundance of neurons was detected in the early stages of organoid generation, compared to the controls, but was not maintained on day 60. Taken together, these results indicate an inappropriately accelerated neuronal differentiation in the L271H condition, as a consequence of the L271H variant. As calcium ions have previously been described to regulate cell cycle and migration [58, 59], these findings suggest a role for the Ca_v_1.3 channel in this neurodevelopmental context. Furthermore, as the cortical organoids serve as a model for the early phases of human corticogenesis [38, 39], dysregulation of ventricular-like structures may suggest impaired development necessary for the subsequent generation of the cortical layers. Interestingly, iPSC-derived models of the Ca_v_1.2 channel in the context of Timothy syndrome have revealed cell-autonomous migration defects explaining impaired corticogenesis in rodent brains and human iPSC-derived organoid models [48, 51, 60]. However, as no gross neuroanatomical alteration are observed in neither patient brains [24] nor mouse models of Ca_v_1.3 variants with similar gating changes [8], these alterations most likely will be restricted to specific substructures of the brain containing specific neural circuits [8] or be masked by compensatory effects in vivo that remain to be investigated. In conclusion, the observed structural disorganization of cortical organoids suggests that the Ca_v_1.3 channel does play a vital role in the very early phases of human brain development and dysregulation of channel function might cause structural abnormalities, ultimately affecting appropriate neural circuitry formation. However, while iPSC-based organoids are powerful, they do not fully recapitulate the structural and functional complexity of the developing human brain, including long-term circuit maturation, behavior, and cell-type diversity. Thus, our findings complement, rather than replace insights gained from animal models [8, 23]. Since *CACNA1D* is also expressed in peripheral tissues [2, 3], and as their dysfunction may also indirectly influence brain development, knock-in animals represent a complementary tool for understanding Ca_V_1.3 dysfunction in a human patient.

Our study identifies transcriptomic alterations in the patient-derived NPCs and neurons providing an unbiased insight into gene dysregulation, that may underlie the complex pathophysiology observed in patients. In total, 78 genes were differentially expressed in the patient-derived NPCs while 173 genes were found to be dysregulated in Ca_v_1.3 L271H-variant neurons. Comparative analysis of the DEGs reported in our study with the SFARI Gene database [61] (Release 2024 Q2) reveals that 17 out of 85 (20%) Ca_v_1.3 L271H-associated upregulated DEGs in the NPCs and neurons are listed in this comprehensive collection of ASD-related genes. This finding suggests that Ca_v_1.3 dysfunction, caused by the L271H mutation, may drive other ASD-related dysregulations. GO analysis highlights changes in processes related to RNA polymerase II-dependent transcription, which suggests that Cav1.3 dysregulation may impact excitation–transcription (E–T) coupling. E–T coupling refers to the process by which electrical or chemical activation of a cell is converted into a signal that reaches the nucleus, controlling gene transcription in an activity-dependent manner [9, 10]. This mechanism is crucial for regulating gene expression, which in turn supports neuronal remodeling. Such remodeling has been reported to be essential for long-term adaptive changes, particularly during neuronal development, learning, memory, and drug addiction [9, 10].

Additionally, GO analysis reveals changes in processes related to “CNS development”, “neuronal differentiation” and “neurogenesis” and several key neurodevelopmental genes were found dysregulated, including *PTN, MEIS2* and *POU3F2 (BRN2)*. *PTN* is essential for the proper development of the human central nervous system [41, 42, 62] and has been shown to promote neurite outgrowth and to enhance neuronal differentiation [63, 64]. Mutation in the homeobox-encoding *MEIS2* gene has been reported to cause syndromic developmental delay, including intellectual disability and facial dysmorphisms such as cleft palate [65, 66]. *BRN2* loss-of-function contributes to impaired positioning of newly produced neurons causing malformation of the neocortex [67] and overexpression of *BRN2* is instrumental for the direct conversion of somatic cells into NPCs and functional neurons [68, 69]. Our finding that Ca_v_1.3 L271H variant may have an impact on these neurodevelopmentally important genes indicates a central role for Ca_v_1.3 at early stages of human neurodevelopment which warrants further investigation. Moreover, our study demonstrates upregulation of *CNTN4*, *CNTNAP2* and *AUTS2* which, besides being associated with ASD, are also risk factors for a range of other neurodevelopmental and neuropsychiatric disorders, including intellectual disability, Tourette syndrome, Schizophrenia, attention deficit hyperactivity disorder (ADHD), and epilepsy. Interestingly, cortical organoids from *AUTS2* patient-derived iPSCs showed aberrant neural rosette structures, caused by reduced apical polarity and alterations in columnar organization [70], which closely resembles the structural alterations observed in the cortical organoids derived from Ca_v_1.3 L271H iPSCs. We hypothesize that Ca_v_1.3 dysregulation during early stages of neurogenesis may result in distorted calcium homeostasis, serving as a common denominator for ASD and other neuropsychiatric disorders. It will be important for future studies to investigate a potential causal relationship between Ca_v_1.3 dysfunction and dysregulation of neurodevelopmentally relevant and neuropsychiatric risk-associated genes such as *CNTN4*, *CNTNAP2* and *AUTS2*. In this context, it is also of interest that, although similar GO terms are enriched for both the NPC and neuronal cultures, the overlap of genes regulating these biological processes is limited. Given the distinct functional properties of NPCs and neurons, this again indicates different roles for Ca_v_1.3 at different stages of neurodevelopment.

Notably, we controlled for genetic background and clonal variability by using five clones of three independent healthy control lines and three subclones of the L271H patient line for a set of experiments. However, to provide a more definitive assessment of variant-specific effects, future studies will require an isogenic line in which the point mutation has been corrected. Although pharmacological rescue using DHPs has been proposed for Ca_v_1.3 gain-of-function variants, the concurrent inhibition of Ca_v_1.2 channels by DHPs complicates interpretation in neural systems where both isoforms are expressed. Future strategies may therefore benefit from combining CRISPR-engineered isogenic lines with selective pharmacological or allele-specific interventions to dissect Ca_v_1.3-specific contributions with higher precision. Additionally, the disease model described here might serve as a platform for in vitro screening of currently unavailable Ca_v_1.3-specific blockers and may thereby pave the way for novel therapeutic strategies aimed at treating Ca_v_1.3 channelopathies.

In conclusion, the Ca_v_1.3 L271H disease model reported in this study provides electrophysiological and transcriptomic evidence that variants in the *CACNA1D* gene can contribute to functional and neurodevelopmental alterations in affected patients. Our research highlights a potential link to other known neurodevelopmental regulators and risk genes for neuropsychiatric disorders, positioning calcium signaling dysregulation as a common cellular factor. Overall, this disease model enhances our understanding of pathophysiological mechanisms underlying the symptoms observed in patients and will be instrumental for the identification of feasible drug targets and the validation of potential pharmaceutical interventions.

## Methods and materials

### iPSC culture

The control (hPSCreg identifiers: JMUi001-B, IBKMOLi006-A, IBKMOLi007-A) or patient-derived *CACNA1D* L271H iPSCs (hPSCreg identifier: IBKMOLi002-A) [26] were cultured (37 °C, 5% CO_2_) on hESC-qualified Matrigel-coated (Corning) plates (stock diluted 1:25 – 1:30 in DMEM/F12, incubated at 4 °C overnight) in StemMACS iPS brew XF (Miltenyi biotech). Media was changed daily. Passaging was performed every 4-5 days (split ratio: 1:10 – 1:20) using Accutase (Sigma Aldrich) and 10 µM ROCK inhibitor (Y-27632, Miltenyi Biotech). For storage, iPSCs were frozen in freezing media, consisting of 80% knock-out serum replacement (ThermoFisher Scientific) and 10% DMSO (Roth), and stored at −80 °C.

### Derivation of NPCs

The control and patient-derived iPSCs were differentiated into small molecule neural progenitor cells (NPCs) according to the protocol previously published by Reinhardt et al. [27]. Briefly, the iPSCs were grown to a confluency of 50–70% and were then dissociated using Accutase (Sigma Aldrich). The cells were transferred into ultra-low-attachment 6-well plates (ThermoFisher Scientific) to induce embryoid body (EB) formation and were cultured in EB formation medium, consisting of DMEM/F12 (ThermoFisher Scientific) supplemented with 20% knockout serum replacement (ThermoFisher Scientific), 100 µM β-mercaptoethanol (ThermoFisher Scientific), 1% non-essential amino acids (Sigma Aldrich), 2 mM GlutaMAX (ThermoFisher Scientific), 10 µM SB431542 (VWR), 1 µM dorsomorphin (Miltenyi Biotec), 3 µM CHIR99021 (Axon Medchem) and 0.5 µM purmorphamine (Miltenyi Biotec).

After EB formation the media was changed to SDCP media consisting of 50% DMEM/F12 (ThermoFisher Scientific) and 50% Neurobasal (ThermoFisher Scientific) supplemented with 0.5% N2 supplement (ThermoFisher Scientific), 1% B27 supplement without vitamin A (ThermoFisher Scientific), 2 mM GlutaMAX (ThermoFisher Scientific), 10 µM SB431542 (VWR), 1 µM dorsomorphin (Miltenyi Biotec), 3 µM CHIR99021 (Axon Medchem) and 0.5 µM Purmorphamine (Miltenyi Biotec). On day 4, the media was exchanged for CPA medium, consisting of 50% DMEM/F12 (ThermoFisher Scientific) and 50% Neurobasal (ThermoFisher Scientific) supplemented with 0.5% N2 supplement (ThermoFisher Scientific), 1% B27 supplement without vitamin A (ThermoFisher Scientific), 2 mM GlutaMAX (ThermoFisher Scientific), 3 µM CHIR99021 (Axon Medchem), 0.5 µM purmorphamine (Miltenyi Biotec) and 150 µM ascorbic acid (Sigma Aldrich).

The EBs were cultured until day 6, where they were mechanically dissociated through trituration, transferred 1:1 to 6-well plates coated with growth-factor-reduced Matrigel (Corning) (stock diluted 1:20 – 1:25 in DMEM/F12, incubated at 4 °C overnight) and continuously cultured in CPA medium. To achieve high purity of the NPCs, the cells were split at high ratios of 1:100 to 1:500 for more than five passages. After purification the cells were continuously cultured on growth-factor-reduced Matrigel (Corning) or Geltrex (ThermoFisher Scientific) coated cell culture plates (stock diluted 1:20 - 1:25 in DMEM/F12, incubated at 4 °C overnight) in CPA medium.

### Immunocytochemistry

Cells were fixed using 4% paraformaldehyde (10 min, 4 °C, Sigma Aldrich) and blocked (1 h, 3% BSA Fraction V, AppliChem), whilst simultaneously being permeabilized using 0.2% Triton X-100 (Sigma Aldrich). Primary antibodies were incubated overnight at 4 °C. Secondary antibodies were incubated for 2.5 h at room temperature. 300 nM DAPI (Carl Roth) was added during the last washing step for nuclear counterstaining. Coverslips were mounted using Aqua-Poly-Mount (Polysciences, Warrington) and were analyzed using a Leica DMi8 fluorescence microscope (Leica). The following antibodies were used: ms-α-NESTIN (R&D systems) Dilution: 1:500, rb-α-PAX6 (BioLegend, San Diego, CA, USA, 1:100, RRID:AB_2565003), RRID:AB_2251304), ms-α-SOX2 (R&D systems, Dilution 1:100, RRID:AB_358009), dky-α-rb-A488 (ThermoFisher, 1:500, RRID:AB_2535792), dky-α-ms-A546 (ThermoFisher, 1:500, RRID:AB_2534012).

### Midbrain neuronal differentiation

Midbrain neurons were differentiated from the control and patient-derived NPCs according to the protocol previously published by Jovanovic and Salti et al., 2018 [35]. Two days before the initiation of the differentiation the NPCs were seeded onto plates coated with a 100 µg/mL solution of poly-L-ornithine (37 °C, overnight) (Sigma Aldrich) and a 10 µg/mL solution (37 °C, 2 h) (Sigma Aldrich) at a density of 2×10^5^ cells per well of a 6-well plate.

The media was changed from CPA medium to midbrain differentiation medium, consisting of 50% DMEM/F12 (ThermoFisher Scientific) and 50% Neurobasal (ThermoFisher Scientific) supplemented with 1% B27 supplement with vitamin A (ThermoFisher Scientific), 2 mM GlutaMAX (ThermoFisher Scientific), 100 ng/mL FGF8 (ThermoFisher Scientific), 1 µM purmorphamine (Miltenyi Biotec) and 200 µM ascorbic acid (Sigma Aldrich), to initiate the differentiation. The cells were split onto freshly coated plates when full confluency was reached (typically day 3) at a density of 1×10^5^ cells per well of a 6-well plate. Maturation of the differentiating cells began on day 8 by switching to medium consisting of 50% DMEM/F12 (ThermoFisher Scientific) and 50% Neurobasal (ThermoFisher Scientific) supplemented with 1% B27 supplement with vitamin A (ThermoFisher Scientific), 2 mM GlutaMAX (ThermoFisher Scientific), 10 ng/mL BDNF (STEMCELL Technologies), 10 ng/mL GDNF (Miltenyi Biotec), 1 ng/mL TGF-β3 (ThermoFisher Scientific), 10 ng/mL BMP5 (R&D systems), 10 ng/mL BMP7 (R&D systems), 500 µM db-cAMP (Sigma Aldrich) and 200 µM ascorbic acid (Sigma Aldrich). Additionally, the medium was supplemented with 0.5 µM purmorphamine (Miltenyi biotec) for the ninth and tenth day of differentiation, before it was withdrawn from the medium.

The differentiating cells typically reached full confluency again on day 13 and were split onto poly-L-ornithine and laminin coated 3.5 cm cell culture dishes (Greiner bio-one) at 1×10^5^ cells per dish to achieve sparse cultures needed for immunocytochemistry and whole-cell patch clamp recordings of individual cells. The cells were cultured (37 °C, 5% CO2) until day 30 prior to analysis. The following antibodies were used: ms-α-TH (Millipore, 1:1000, RRID:AB_2201528), rb-α-TUBB3 (Abcam, Cambridge, UK, 1:500, RRID:AB_869991), dky-α-rb-A488 (ThermoFisher, 1:500, RRID:AB_2535792), dky-α-ms-A546 (ThermoFisher, 1:500, RRID:AB_2534012).

### Calcium imaging

For the NPCs, three subclones from patient-derived *CACNA1D* L271H iPSCs (derived hPSCreg identifier: IBKMOLi002-A) were used and compared to five control lines (derived from hPSCreg identifiers: JMUi001-B, IBKMOLi006-A, IBKMOLi007-A) in six independent culture preparations. For neurons, the L271H line (hPSCreg identifier: IBKMOLi002-A) and one independent control line (hPSCreg identifiers: JMUi001-B) were analyzed in one culture preparation. Calcium imaging of NPCs and midbrain neurons was performed blinded regarding the sample condition. NPCs and neurons were loaded for 30 min at 37 °C with 1 μM Calbryte 520AM (AAT Bioquest) in low KCl Tyrode’s solution (5 mM KCl, 129 mM NaCl, 2 mM CaCl_2_, 1 mM MgCl_2_, 30 mM glucose, and 25 mM HEPES, pH 7.4, supplemented with 0.02% Pluronic-F27). Midbrain neurons were loaded in the maturation medium. Cells were incubated for 10 min at RT to allow de-esterification of the dye and washed with Tyrode’s solution. Fluorescent signals were recorded with cellSens software (Olympus, Japan) at a 2.6 Hz frame rate using an Orca Fusion BT digital CMOS camera (Hamamatsu, Japan) connected to a BX53 microscope (Olympus) equipped with a 40× water immersion lens (NA 0.8). Time-lapse image sequences of at least 3 fields of views per condition were acquired for 10–20 min at room temperature. The image series were further processed using ImageJ. First, a region-of-interest (ROI) was manually placed around each cell’s soma. Subsequently, mean grey values of each ROI in each frame were measured using the ROI manager tool. Background fluorescence was subtracted and values were normalized to baseline fluorescence (ΔF/F0, with F0 being the minimal signal for a ROI and ΔF being the difference between F0 and a signal at a given time point). Calcium events were manually categorized as either calcium waves (slow rising phases, ∆F/F < 0.2) or spikes (fast-rising phase events, ∆F/F > 0.2) according to previously published definitions [29, 71].

Distribution of datasets was evaluated with SigmaPlot (Systat Software) and a Mann–Whitney U test was applied for skewed datasets. A chi-square (Χ²) test was used for categorical data. Significance criteria were *P  <  0.05, **P  <  0.01 and ***P  <  0.001. Graphs depict violin plots with median and quartiles. Data and graphs were organized and analyzed using ImageJ, Microsoft Excel, GraphPad Prism 8.3 and SigmaPlot (Systat Software).

### Whole-cell current-clamp recordings

The L271H line (hPSCreg identifier: IBKMOLi002-A) and one control line (hPSC-reg identifier: JMUi001-B) were analyzed in two (NPCs) and three (neurons) independent culture preparations. Action potentials (APs) of individual NPCs and midbrain neurons were recorded in the current-clamp configuration of the whole-cell mode. Recordings were performed blinded to reduce potential biases. Patch pipettes were filled with 130 mM K-Gluconate, 1 mM MgCl_2_, 10 mM HEPES, 5 mM EGTA, 4 mM Mg-ATP, and 300 μM Na-GTP (pH 7.2 with KOH). The bath solution contained 137 mM NaCl, 3 mM KCl, 10 mM HEPES, 2 mM MgCl_2_, 1.8 mM CaCl_2_, and 10 mM Glucose (pH 7.4 with NaOH). Recordings were performed at room temperature using an EPC-10 amplifier (HEKA Elektronik) controlled by the PatchMaster software (v.2.80, HEKA Elektronik). The data were sampled at 10 kHz and low-pass filtered at 2.9 kHz. Spontaneous action potentials were recorded for a period of >1 min without injecting any current. To determine the threshold of action potential firing and spiking response, the resting membrane potential was clamped to −70 mV by current injection to stop spontaneous activity. Subsequently, pulses of increasing intensity were applied (2 s, 0–10 pA). Data analysis of action potential parameters was performed using the Clampfit software (v.10.7, Axon Instruments). Action potential classification was based on previously published definitions [71–75].

Distribution of datasets was evaluated with SigmaPlot (Systat Software). For normally distributed datasets, statistical significance was calculated using unpaired Student’s t test while the Mann–Whitney U test was applied for skewed datasets, with significance criteria *P  <  0.05, **P  <  0.01 and ***P  <  0.001. A chi-square (Χ²) test was used for categorical data (firing modes). Graphs either depict scatter dot plots showing values of individual cells (dots) and means ± SEM, or violin plots with median and quartiles (skewed datasets). Data and graphs were organized and analyzed using Microsoft Excel, GraphPad Prism 8.3 and SigmaPlot (Systat Software).

### Generation of cortical organoids

Cortical organoids were generated from one control (hPSC-reg identifier: JMUi001-B) and the L271H-iPSC line (hPSCreg identifier: IBKMOLi002-A) following an adapted protocol of Lancaster and Knoblich, 2014, as well as Lancaster *et al*., 2018 [38, 39]. Four independent batches of organoids were generated and analyzed at D10, D20, D30 (batch #1-3) and D60 (batch #4).

The iPSCs were cultured in StemMACS iPS brew (Miltenyi Biotech) on hESC-qualified Matrigel-coated (Corning) 6-well plates until they reached a confluency of 70–80%. The cells were harvested using Accutase (ThermoFisher Scientific), counted and transferred to a U-bottom ultralow attachment 96-well plate (Corning). In each well, 9×10^4^ cells were seeded in the presence of 10 µM ROCK inhibitor (Y27632, Miltenyi Biotech), allowing for the subsequent EB formation. After 72 h of incubation, correct EB formation was verified, and half the media was changed.

Neural induction began on day 6, when medium was changed from iPS brew to neural induction medium (NIM), consisting of DMEM/F12 (ThermoFisher Scientific), 1% N2 supplement (ThermoFisher Scientific), 2 mM GlutaMAX (ThermoFisher Scientific), 1% non-essential amino acids (Sigma Aldrich) and 1 ng/mL heparin solution (Merck). The medium was sterile-filtered over a 0.22 µm filter before use. Half the medium was changed on days 7, 8 and 9.

The growing aggregates were embedded into 1% liquid Matrigel (Corning) dispersed in NIM medium on day 10 and manually transferred to 10 cm cell culture dishes (24 aggregates per dish), which had been rinsed with anti-adherence solution (STEMCELL Technologies) and washed with 1x PBS (ThermoFisher Scientific).

On day 13, the NIM medium was replaced by improved organoid differentiation medium without vitamin A (IMP-A), consisting of 50% DMEM/F12 (ThermoFisher Scientific), 50% Neurobasal (ThermoFisher Scientific), 1% B27 -A (ThermoFisher Scientific), 0.5% N2 supplement (ThermoFisher Scientific), 2 mM GlutaMAX (ThermoFisher Scientific), 0.5% non-essential amino acids (Sigma Aldrich), 10 ng/mL insulin solution (Sigma Aldrich) and 1% antibiotic-antimycotic solution (ThermoFisher Scientific). Additionally, 3 µM CHIR99021 (Axon Medchem) were added to the media on day 13 and 14. Media changes with IMP-A were also performed on days 16, 19 and 22. From day 19 onwards the dishes containing the floating aggregates were cultured on an orbital shaker (58 rpm) placed inside the incubator.

Between day 25 and day 60 the aggregates were cultured in improved organoid differentiation medium with vitamin A (IMP + A), consisting of 50% DMEM/F12 (ThermoFisher Scientific), 50% Neurobasal (ThermoFisher Scientific), 1% B27 + A (ThermoFisher Scientific), 0.5% N2 supplement (ThermoFisher Scientific), 2 mM GlutaMAX (ThermoFisher Scientific), 0.5% non-essential amino acids (Sigma Aldrich), 10 ng/mL insulin solution (Sigma Aldrich), 0.05 g sodium bicarbonate (ThermoFisher Scientific), 55 µM β-mercaptoethanol, 400 µM ascorbic acid and 1% antibiotic-antimycotic solution (ThermoFisher Scientific). The medium was changed every 3-4 days.

On days 10, 20, 30 and 60, three organoids each from the control and L271H lines were harvested for staining. The organoids were manually transferred into a 2 mL centrifugation tube and washed with 1x PBS, before they were fixed in a 4% paraformaldehyde solution (Sigma Aldrich) for 30 min to 1.5 h at room temperature on an orbital shaker. Subsequently, the organoids were washed three times in 1x PBS (10 min each, room temperature, orbital shaker) before being transferred to a 30% sucrose solution (Sigma Aldrich), in which they were incubated for at least 24 h at 4 °C. The organoids were embedded in O.C.T compound (Tissue-Tek) and frozen at −80 °C.

The frozen organoids were cut into a series of 20 µm sections using a cryotome and subsequently stained. A post-fixation step with a 4% paraformaldehyde solution (Sigma Aldrich) was performed for 15 min. Each slice was washed twice (5 min each) using 1x PBS and washed once using PBS-T (0.2% Triton X-100 in PBS, Sigma Aldrich). The slices were incubated in blocking solution, consisting of 3% BSA fraction V (AppliChem) in PBS-T for 1.5–2 h. Primary antibodies were diluted in blocking solution and incubated overnight at 4 °C. Slides were washed three times with PBS-T (5 min each, RT) before the secondary antibodies were applied. Secondary antibodies were diluted in 1.5% BSA/PBS-T and added for 1–1.5 h at room temperature. Nuclear counterstaining was performed with DAPI (10 min, RT). Subsequently, the slices were washed twice with PBS-T and once with PBS before they were sealed using Aqua-Poly-Mount (Polysciences). Stainings were imaged on a Leica DMi8 fluorescence microscope. The following antibodies were used: ms-α-MAP2 (Sigma Aldrich, 1:500, RRID:AB_477193), rb-α-PAX6 (BioLegend,1:500, RRID:AB_2565003), ms-α-pVIM (MBL international, 1:500, RRID:AB_592962), ms-α-SOX2 (R&D system, 1:100, RRID:AB_358009), rb-α-TBR2 (abcam, 1:100, RRID:AB_778267), dky-α-rb-A488 (ThermoFisher, 1:500, RRID:AB_2535792), dky-α-ms-A488 (ThermoFisher, 1:500, RRID:AB_2535792), dky-α-ms-A546 (ThermoFisher, 1:500, RRID:AB_2534012), dky-α-rb-A594 (ThermoFisher, 1:500, RRID:AB_141637).

### Image analysis

The size of cortical organoids was assessed at days 3, 6, 10 and 20 by measuring the area of the aggregates. Epifluorescence pictures were taken using a Leica DMi8 microscope with constant parameters and analyzed using FIJI (v1.52-1.54p). To quantify the area covered by SOX2, pVIM and MAP2 signal, pictures were taken with the same settings randomly across the sections. The positive area of the section was measured for each marker after applying the same threshold to each picture and normalizing the resulting values to the average value of the area of each picture covered by DAPI. To quantify VS size and number, VS were identified by positive expression of neural progenitor markers PAX6 and SOX2 and the area was measured. pVIM+ and TBR2+ cells were counted manually and normalized to the DAPI area. To assess the percentage of pVIM+ cells inside or outside of VZ, cells were first manually counted and assigned to either the VZ area or the area outside the rosette of 100. Data were statistically analyzed with Microsoft Excel and GraphPad Prism 6.0. All the results are presented as the mean ± standard deviation and individual data points are displayed. Statistics were calculated using unpaired Student’s t-test or one-way ANOVA with Tukey’s multiple comparisons correction.

### Bulk RNA sequencing

The RNA of control (hPSC-reg identifier: JMUi001-B) and L271H iPSCs, NPCs and midbrain neurons (hPSCreg identifier: IBKMOLi002-A) was isolated from triplicate cultures (biological replicates) using the RNeasy Kit (Qiagen) by following the manufacturer’s protocol. To generate cDNA libraries, 500 ng of each RNA sample were used in combination with the “QuantSeq 3’ mRNA-Seq V2 Library Prep Kit with UDI” (Lexogen), allowing for dual i5 and i7 indexing of the transcripts. The cDNA synthesis was carried out according to the manufacturer’s protocol. The obtained cDNA library was purified using magnetic beads (Lexogen). Subsequently, a unique combination of i5 and i7 primers were added to each sample to achieve dual indexing of the transcripts. The library was amplified in a RT-qPCR thermocycler (BioRad, Hercules, CA, USA) in the presence of EvaGreen dye to monitor the rate of amplification and to prevent over-amplification of the library. The amplified libraries were subsequently purified using magnetic beads (Lexogen). Ultimately, the libraries were pooled into individual sequencing samples and diluted to a final concentration of 5 ng/μL for next-generation Illumina sequencing.

The computational results presented here have (in part) been achieved using the LEO high performance-computing infrastructure of the University of Innsbruck. The fastq-files, obtained after index demultiplexing performed by the sequencing facility, were used to perform transcript count analysis. To this end, the UMI-tools package (v.1.1.2) was used to extract the unique barcodes from each read [76]. Subsequently, the Bbduk package of the bbmap suite (v.39.01, Bushnell B. – available at: sourceforge.net/projects/bbmap/) was used to trim low quality sequencing tails, polyA-readthroughs and illumine sequencing adapter contaminations. The FastQC package was used to monitor read quality before and after trimming (Andrews et al., available at: http://www.bioinformatics.babraham.ac.uk/projects/fastqc). The reads were then aligned to the reference genome (Ensembl GRCh38 Homo sapiens primary assembly, release 109) using the STAR aligner packager (v.2.7.10b) with settings modified to match the library specifics [77]. After read mapping the UMI-tools package was used to collapse reads with identical UMIs and identical mapping coordinates, to remove PCR duplicates. Final quality control was performed using the RSeQC package (v.5.0.1) to determine the distribution of reads mapped to exons, introns and 3’- or 5’- untranslated regions [78]. Lastly, HTSeq-count (v2.0.2) was used to perform read counting to generate the expression matrix for each sample [79]. As the Ca_v_1.3 L271H and control lines were derived from a female or male donor, respectively, transcripts mapped to genes located on the X and Y chromosomes were removed prior to the analysis. Variance stabilizing transformation was conducted and normalized gene expression matrices were generated. Differential gene expression analysis was performed in R (v4.2.2) using the DESeq2 package [80]. Differentially expressed genes were considered significant if the log_2_FoldcChange was greater than 0.5 or lower than −0.5 and the corresponding adjusted P-Value (Bonferroni correction for multiple testing) was lower than 1 × 10^−4^. Volcano plots of the differentially expressed genes were generated using the EnhancedVolcanao package (v.1.14.0) [81]. Gene ontology (GO) and network analysis was performed using the ShinyGO application (v.0.76) [82].

## Supplementary information

Supplementary figure legends

Supplementary Figure 1

Supplementary Figure 2

Supplementary Figure 3

Suppl. Video1

Suppl. Video2

Supplementary Table

## Data Availability

Due to the sensitivity of the RNA sequencing data, the data will be made available upon reasonable request. Please contact the corresponding author directly.
