# Supplementary material for: Aberrant calcium signaling and neuronal activity in the L271H CACNA1D (Cav1.3) iPSC model of neurodevelopmental disease
Source: Mol Psychiatry. 2026 Jan 9;31(5):2927–40. doi: 10.1038/s41380-025-03429-8 (PMC13099384; doi:10.1038/s41380-025-03429-8)
Supplement: Supplementary file 1 — Supplementary figure legends [file 41380_2025_3429_MOESM1_ESM.docx]

**Supplementary information**

Supplementary Information is available at MP’s website. This includes the following files:

**Suppl. Table 1:** Parameters defining the action potential shape in CTRL and L271H midbrain neurons during induced electrical activity

**Suppl Table 2:** DEG and GO term analysis of CTRL and L271H iPSCs, NPCs, and midbrain neurons.

**Suppl. Table 3:** DEG analysis of ion channel subunits in CTRL and L271H iPSCs, NPCs, and midbrain neurons.

**Suppl. Video 1:** Calcium imaging recording of CTRL and L271H NPCs

**Suppl. Video 2:** Calcium imaging recording of CTRL and L271H midbrain neurons

**Suppl. figure legends**

**Suppl. Figure 1: Validation of midbrain neuron identity**

**A** Normalized count of *CACNA1C* transcripts at various stages of *in vitro* differentiation. * = adj. P < 0.05, **** = adj. P < 0.0001 **B** Combined normalized count of *CACNA1C* and *CACNA1D* transcripts at various stages of *in vitro* differentiation. * = P < 0.05, ** = P < 0.01 **C** Expression levels of TH, FOXA2, EN1, and LMX1B in CTRL and L271H iPSCs, NPCs and midbrain neurons. **D, E** Immunostainings of TUBB3, EN1, and DDC of CTRL and L271H midbrain neurons after 30 days of differentiation. Scale bars = 50 µm.

**Suppl. Figure 2: Size measurements of organoids and ventricular-like structures**

**A** Diameter of CTRL and L271H organoids at days 3, 6, 10 and 20. N = 18 pictures (6 pictures each from 3 independent batches) per condition per timepoint. One-way ANOVA with Tukey’s multiple comparison test. *** = P < 0.001 **B-D** Area, count and size of ventricular-like structures in the day 20 CTRL and L271H organoids. N = 10-11 organoids from three independent batches per condition. Unpaired t-test, * = P < 0.05; **** = P < 0.0001 **E-G** Quantification of pVIM+ radial glial cell abundance and distribution inside or outside of ventricular-like structures (VS) in day 30 organoids. Unpaired t-test, *** = P <0.001 **H** Representative images of TBR2/pVIM immunostainings at various time-points of organoid generation.

**Suppl. Figure 3: GO term analysis of downregulated DEGs**

**A, B** KEGG pathways and GO Biological processes downregulated in NPCs affected by the Ca_v_1.3 L271H mutation. **C, D** KEGG pathways and GO Biological processes downregulated in neurons affected by the Ca_v_1.3 L271H mutation.
