# Supplementary figures and images for: Aberrant calcium signaling and neuronal activity in the L271H CACNA1D (Cav1.3) iPSC model of neurodevelopmental disease
Source: Mol Psychiatry. 2026 Jan 9;31(5):2927–40. doi: 10.1038/s41380-025-03429-8 (PMC13099384; doi:10.1038/s41380-025-03429-8)

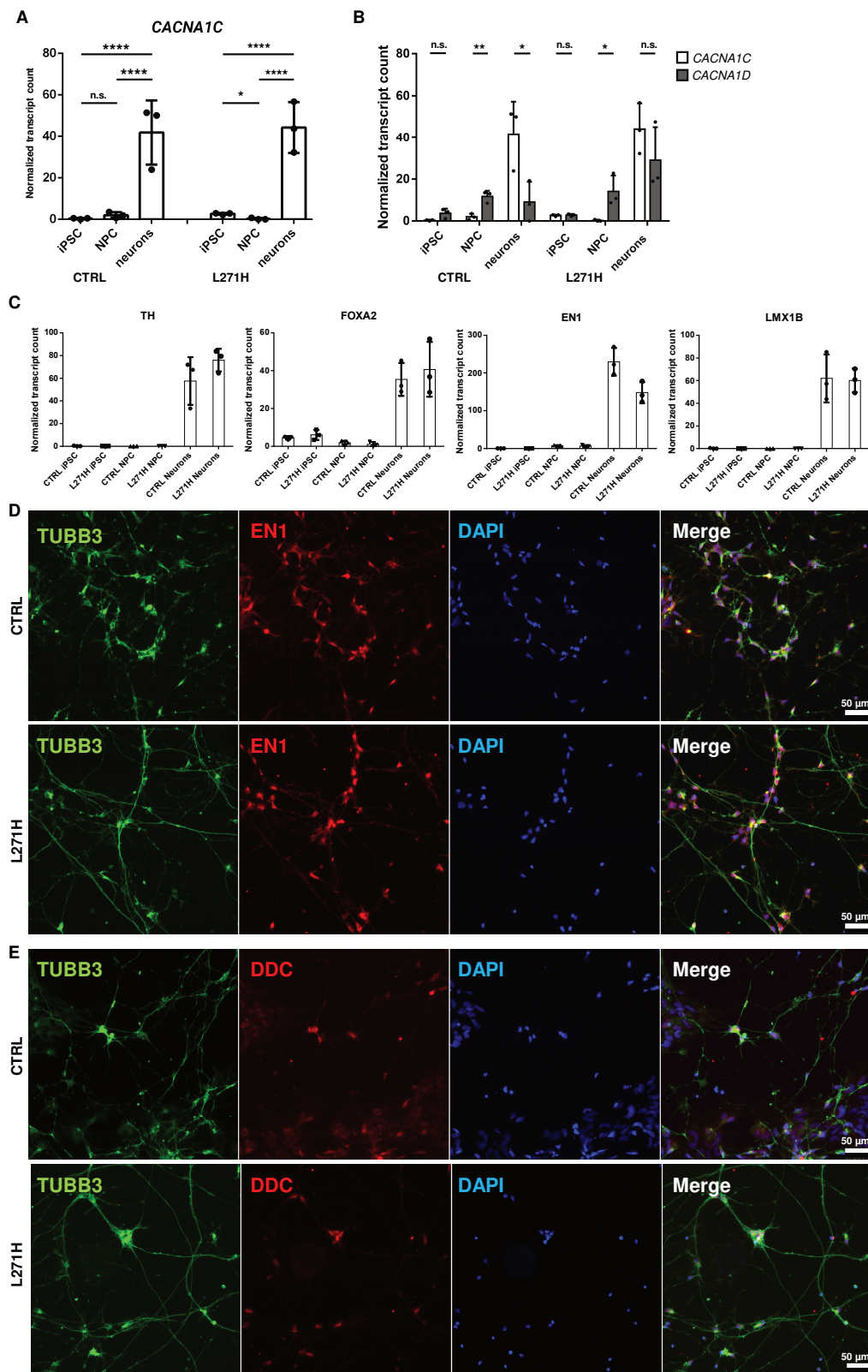

Supplement: Supplementary file 2 — Supplementary Figure 1 [file 41380_2025_3429_MOESM2_ESM.pdf]

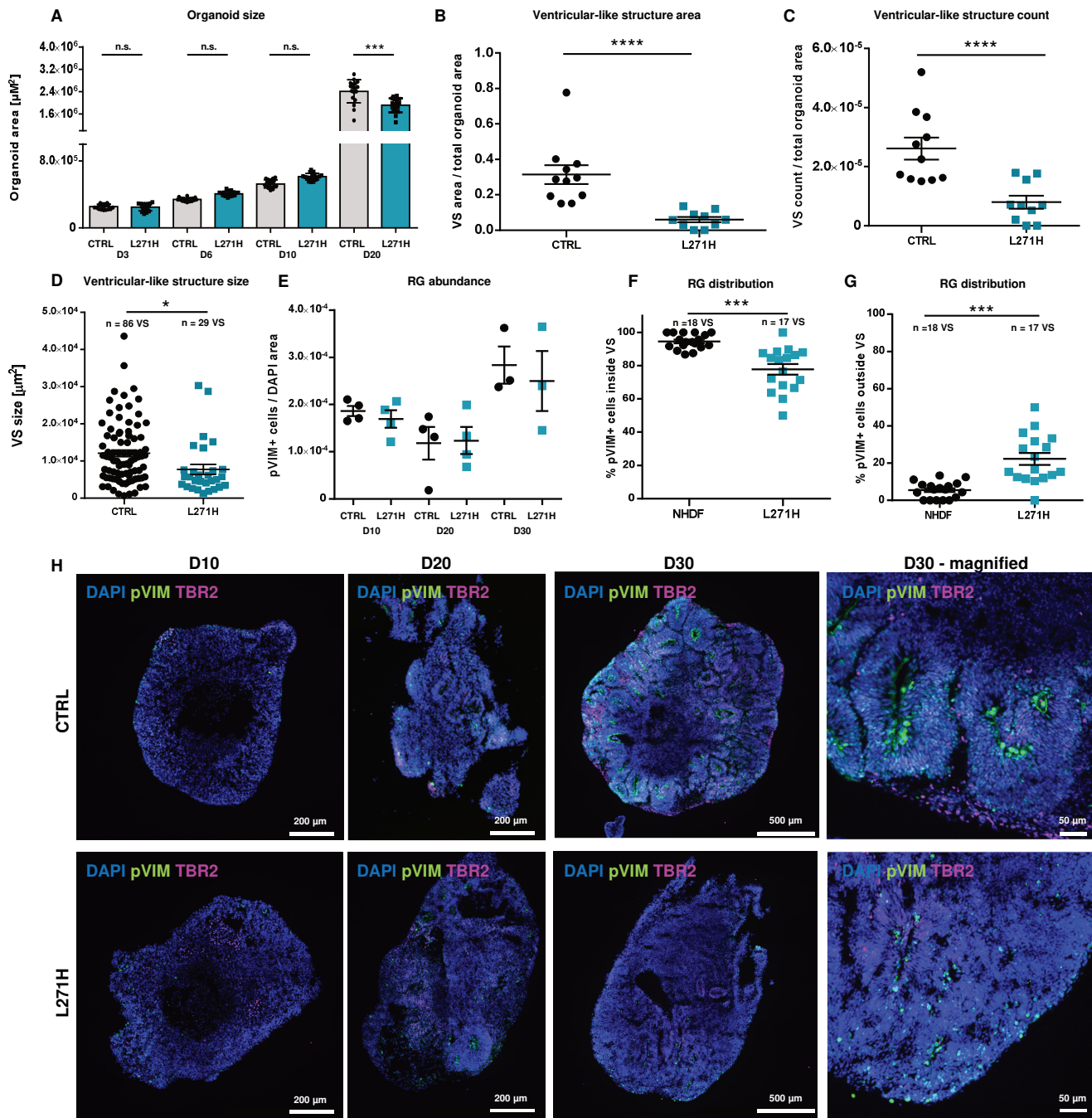

Supplement: Supplementary file 3 — Supplementary Figure 2 [file 41380_2025_3429_MOESM3_ESM.pdf]
