# Supplementary material for: Aberrant calcium signaling and neuronal activity in the L271H CACNA1D (Cav1.3) iPSC model of neurodevelopmental disease
Source: Mol Psychiatry. 2026 Jan 9;31(5):2927–40. doi: 10.1038/s41380-025-03429-8 (PMC13099384; doi:10.1038/s41380-025-03429-8)
Supplement: Supplementary file 4 — Supplementary Figure 3 [file 41380_2025_3429_MOESM4_ESM.pdf]

Downregulated DEGs L271H NPCs

A **KEGG**

Enrichment FDR	nGenes	Pathway Genes	Fold Enrichment	Pathways (click for details)
1.8E-02	3	193	19.8	Transcriptional misregulation in cancer

B **GO biological process**

Enrichment FDR	nGenes	Pathway Genes	Fold Enrichment	Pathways (click for details)
1.3E-02	3	95	40.1	Embryonic skeletal system morphogenesis
1.3E-02	3	99	38.5	Negative reg. of myeloid cell differentiation
1.9E-02	3	130	29.3	Embryonic skeletal system development
1.2E-02	4	233	21.8	Reg. of myeloid cell differentiation
1.5E-02	4	312	16.3	Embryonic organ morphogenesis
1.2E-02	5	480	13.2	Embryonic organ development
3.6E-02	4	450	11.3	Myeloid cell differentiation
3.6E-02	4	457	11.1	Reg. of hemopoiesis
3.6E-02	6	1316	5.8	Positive reg. of transcription by RNA polymerase II

Downregulated DEGs L271H Neurons

C **KEGG**

Enrichment FDR	nGenes	Pathway Genes	Fold Enrichment	Pathways (click for details)
1.2E-08	16	354	8.1	PI3K-Akt signaling pathway
7.3E-11	15	200	13.4	Focal adhesion
6.9E-06	15	530	5.1	Pathways in cancer
7.6E-06	12	331	6.5	Human papillomavirus infection
8.8E-08	9	88	18.3	ECM-receptor interaction
2.7E-07	9	103	15.6	Protein digestion and absorption
1.6E-06	8	92	15.5	Small cell lung cancer
3.0E-06	8	102	14	Amoebiasis
2.4E-04	8	202	7.1	Proteoglycans in cancer
2.8E-05	7	100	12.5	AGE-RAGE signaling pathway in diabetic complications
4.1E-04	7	161	7.8	MicroRNAs in cancer
5.7E-05	6	73	14.7	P53 signaling pathway
1.8E-04	6	90	11.9	Hypertrophic cardiomyopathy
2.3E-04	6	96	11.2	Dilated cardiomyopathy
9.8E-04	6	129	8.3	Relaxin signaling pathway
7.2E-03	6	193	5.6	Transcriptional misregulation in cancer
1.2E-02	6	217	4.9	Reg. of actin cytoskeleton
1.3E-02	6	222	4.8	Human T-cell leukemia virus 1 infection
1.9E-03	5	93	9.6	TGF-beta signaling pathway
6.6E-03	5	124	7.2	Platelet activation

D **GO Biological process**

Enrichment FDR	nGenes	Pathway Genes	Fold Enrichment	Pathways (click for details)
4.9E-20	77	4617	3	System development
4.2E-19	75	4552	2.9	Cell differentiation
4.9E-19	75	4576	2.9	Cellular developmental proc.
6.2E-25	68	2867	4.2	Anatomical structure morphogenesis
5.3E-18	67	3774	3.2	Animal organ development
1.1E-10	63	4821	2.3	Response to chemical
1.9E-09	57	4338	2.3	Reg. of response to stimulus
5.9E-22	56	2086	4.8	Tissue development
5.8E-13	55	3269	3	Response to organic substance
2.5E-11	55	3615	2.7	Reg. of signaling
6.8E-11	54	3602	2.7	Reg. of cell communication
9.6E-08	54	4424	2.2	Response to stress
4.8E-11	51	3210	2.8	Reg. of signal transduction
1.2E-10	51	3300	2.8	Cellular response to chemical stimulus
5.3E-11	49	2990	2.9	Cell surface receptor signaling pathway
4.7E-12	48	2680	3.2	Reg. of developmental proc.
2.7E-10	48	3024	2.8	Reg. of multicellular organismal proc.
1.1E-10	45	2609	3.1	Cellular response to organic substance
1.0E-12	44	2143	3.7	Cell population proliferation
2.2E-04	43	4103	1.9	Reg. of biological quality
